# An atlas of mitochondrial ATP synthase activity across the lifespan

**DOI:** 10.1016/j.molmet.2025.102278

**Published:** 2025-11-01

**Authors:** Muzna Saqib, Dylan C. Sarver, Christy M. Nguyen, Fangluo Chen, Marcus M. Seldin, G. William Wong

**Affiliations:** 1 Department of Physiology, Pharmacology and Therapeutics, Johns Hopkins University School of Medicine, Baltimore, MD, USA; 2 Center for Metabolism and Obesity Research, Johns Hopkins University School of Medicine, Baltimore, MD, USA; 3 Department of Biological Chemistry, University of California, Irvine, USA; 4 Center for Epigenetics and Metabolism, University of California Irvine, Irvine, USA

**Keywords:** ATP synthase activity, Mitochondria, Aging, Sex differences

## Abstract

Mitochondrial dysfunction and declining energy production are hallmarks of aging, yet we lack a comprehensive systems-level view of ATP synthase (Complex V) activity across tissues, sex, and age. To overcome this, we leveraged a recently developed method to directly quantify complex V hydrolytic activity at scale in 32 tissues from young (10 weeks) and old (80 weeks) male and female mice. Our high-resolution atlas reveals several notable findings: 1) complex V activity differs markedly across tissues, with the highest levels seen in contractile organs such as the heart and striated muscles (quadriceps, hamstring, diaphragm, tongue); 2) sex influences complex V activity in a tissue-specific manner, with significant differences seen in the heart, liver, fat depots, pancreas, spleen, tongue, and cortex; 3) aging has a much larger impact than sex on complex V activity, with a greater number of age-dependent changes seen across tissues; 4) the directionality and magnitude of change in complex V activity across sex and age is variable and tissue dependent; 5) the expression of complex V related genes in human and mouse tissues across age shows only partial concordance with complex V activity, suggesting functional modulation by posttranscriptional mechanisms. This compendium of ATP synthase activity highlights organ-level variations in the mode and tempo of aging, affording an unprecedented view of the shared and divergent changes in ATP synthase function across sex and organ systems. Our data provide a valuable reference for comparative studies of mitochondrial adaptations across space and time, and in pathophysiological contexts.

## Highlights

• ATP synthase activity in 32 tissues across sex and the lifespan.
• Sex influences ATP synthase activity in a tissue-specific manner.
• Aging has a much larger impact than sex on ATP synthase activity.
• The directionality and magnitude of change in ATP synthase activity across age is sex- and tissue-dependent.
• Expression of ATP synthase genes shows only partial concordance with activity.

## 1 Introduction

Aging appears to be a universal biological phenomenon with complex underlying causes [1]. Mitochondrial dysfunction is known to be a hallmark of aging and a major driver of functional decline across organ systems [2,3]. Central to mitochondrial energy metabolism is ATP synthase (Complex V), a multi-subunit rotary enzyme complex that harnesses the proton motive force to generate ATP from ADP and inorganic phosphate [4]. With advancing age, its efficiency is thought to decrease, leading to impaired energy production, metabolic inflexibility, and increased oxidative stress [[5], [6], [7]]. Understanding how ATP synthase activity is regulated in different tissues and physiological contexts is essential for elucidating changes that accompany organismal aging.

In energy-demanding tissues, most ATP is produced through oxidative phosphorylation, in which the electron transport chain establishes an electrochemical proton gradient across the inner mitochondrial membrane that is then used to drive the rotary catalysis of ATP synthase [8]. Beyond this canonical role, ATP synthase also functions as a bidirectional nanomachine: when the proton motive force (PMF) collapses, it can reverse its cycle, hydrolyzing ATP to pump protons back into the intermembrane space to restore the proton gradient and preserve mitochondrial membrane potential [9,10]. This reversibility serves to support redox balance and safeguards mitochondrial integrity during energetic stress, thus making ATP synthase activity one of the most sensitive indicators of mitochondrial health and dysfunction [11].

Despite its central role in cellular bioenergetics, surprisingly little is known about how ATP synthase activity is modulated by sex and age across diverse organs and tissues. Sex is known to influence mitochondrial physiology, such as oxidative phosphorylation efficiency, reactive oxygen species handling, and metabolic flexibility [12,13]. Accordingly, sex differences contribute to divergent risks for cardiovascular, metabolic, and neurodegenerative disorders [14]. Age is another major factor affecting mitochondrial health and function, with marked differences in the rate and trajectory of aging seen across sex and tissues [[15], [16], [17]]. However, how sex and age interact to influence ATP synthase activity at the systems level is largely unknown. In this study, we aim to uncover shared and divergent signatures of ATP synthase function across age, sex, and organ systems.

We have previously leveraged a novel frozen-tissue approach [18] to generate a high-resolution mitochondrial respiration atlas [19]. In this study, we adapted a novel approach recently developed by Fernandez-Del-Rio and co-workers to quantify ATP synthase activity by measuring the maximal hydrolytic capacity of the enzyme complex [20]. The hydrolysis of ATP through the reverse mode of ATP synthase produces protons that acidify the medium, and the acidification rate (AR) as measured by the Seahorse XF Analyzer serves as a surrogate for ATP synthase activity [20]. This approach allows us to directly and efficiently measure ATP synthase hydrolytic capacity at scale in frozen tissue homogenates in a standardized manner across 32 tissues from 40 mice without the need for mitochondrial isolation. Our study consisted of 1280 tissue samples and 3840 assays with three technical replicates. The large-scale data provide an unprecedented systems-level view of ATP synthase activity across tissues, sex, and age.

We show that while sex clearly influences ATP synthase activity across tissues, age has a proportionately larger impact. While many tissues show a decline in ATP synthase activity with age, some tissues have elevated activity, possibly reflecting a compensatory response to altered mitochondrial function with age. Similar to what we have shown for mitochondrial respiration [19], ATP synthase activity in some tissues is remarkably resilient to age-induced changes. The global view of shared and divergent changes in ATP synthase activity across the lifespan underscores the complex and heterogenous responses of male and female tissues to the aging process. Our data lend further support to recent large-scale transcriptomic and proteomic studies highlighting variations in the mode and tempo of aging across sex and tissues in humans and mice [15,[21], [22], [23]], and provide a valuable resource for future studies of mitochondrial adaptation in health and disease.

## 2 Materials and methods

### 2.1 Mouse model

All wild-type C57BL/6J male and female mice were purchased from the Jackson Laboratory and fed a standard chow (Envigo; 2018SX). In total, the young group was comprised of 10 male and 10 female 10-week-old mice, and the old group was comprised of 10 male and 10 female 80-week-old mice. Mice were housed in polycarbonate cages on a 12h:12h light–dark photocycle with ad libitum access to water and food. All mice were fasted for 2 h prior to euthanasia and dissection. Tissues were collected, snap-frozen in liquid nitrogen, and kept at −80 °C until analysis. All mouse protocols were approved by the Institutional Animal Care and Use Committee of the Johns Hopkins University School of Medicine (animal protocol # MO22M367). All animal experiments were conducted in accordance with the National Institute of Health guidelines and followed the standards established by the Animal Welfare Acts.

### 2.2 Comprehensive multi-organ dissection

**Figure 1:**
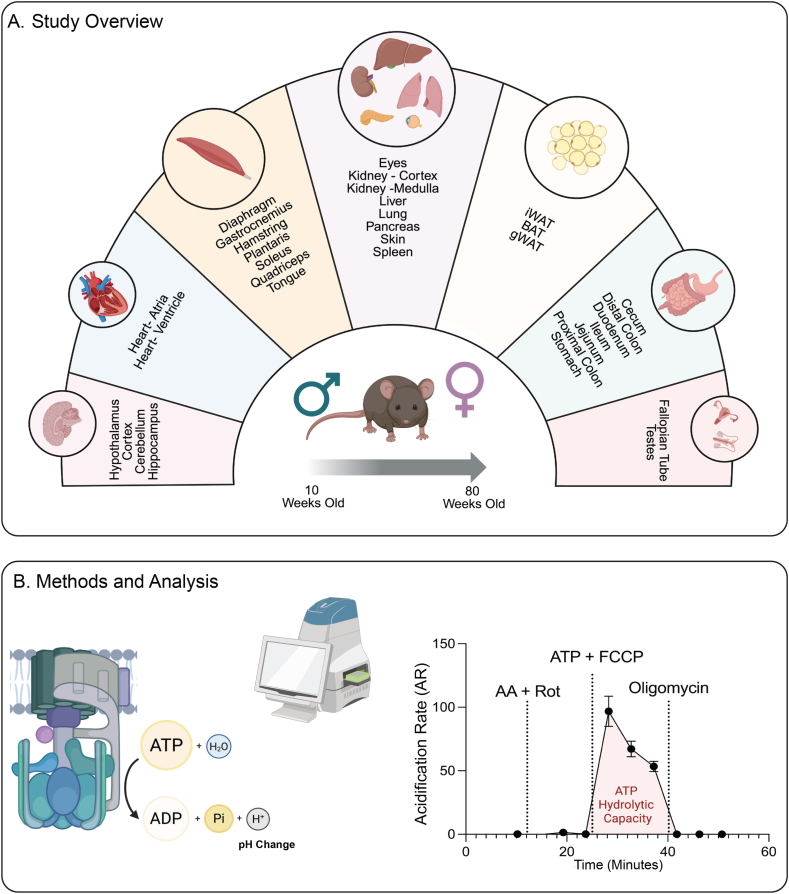
**Overview of workflow and analysis pipeline for ATP synthase activity across tissues.** (A) A diagram detailing the collection of 32 different tissues from four groups of mice (*n* = 10 per group). The four groups consist of young males, young females, old males, and old females, with young defined as 10-week-old and old as 80-week-old. (B) A schematic of ATP synthase highlighting the key components of the assay used to evaluate ATP synthase activity, as well as the type of data and output obtained post analysis.

Multi organ dissections are outlined in Figure 1A. We used the same tissues collected in our recent study [19]. Each mouse was dissected cleanly, swiftly, and in a concerted manner by three people. Each mouse dissection took approximately 8–10 min, with 32 tissues collected per dissection. After euthanasia, blood was collected via decapitation. The head was then immediately given to dissector one to collect brain regions (hypothalamus, cerebellum, hippocampus, and cortex), eyes, and tongue. Simultaneously, the visceral cavity was opened by dissector two. Inguinal white adipose tissue (iWAT) was collected immediately after opening the abdominal skin. After that the abdominal muscle was cut and gonadal white adipose tissue (gWAT) and testes or fallopian tubes were collected. Following this, the visceral organs were partitioned in two groups. Dissector two promptly dissected liver, stomach, kidneys (further separated the cortex and medulla), spleen, diaphragm, heart (further divided into atrium and ventricles), lungs, brown adipose tissue (BAT), and skin (cleared of hair using Topical Nair lotion, cleaned, then collected). At the same time, dissector three was collecting the pancreas, small intestine (further split into duodenum, jejunum, and ileum), cecum, and large intestine (further split into proximal and distal colon). As soon as tissue collection in the head was finished, the mouse carcass was cut transversely at the lumbar spine and handed to dissector one for muscle dissection. Dissector one then rapidly and precisely anatomized (bilaterally) the quadriceps (entire complex – rectus femoris, vastus lateralis, vastus intermedius, and vastus medialis), hamstrings (biceps femoris), gastrocnemius, plantaris, and soleus muscles. All tissues were washed with sterile 1X PBS to remove residual blood prior to snap freeze. Additionally, the stomach, small intestine, and large intestine were cleared and cleaned of debris with PBS prior to freezing. The Cecum, however, was kept whole containing all fecal/food matter and microorganisms present. All dissected tissues were snap frozen in liquid nitrogen and stored at −80 °C for later analysis.

### 2.3 Assay to measure ATP hydrolytic activity of complex V

ATP hydrolytic activity of Complex V (CV) was assayed on a Seahorse XFe96 Analyzer as described in details by Fernandez-Del-Rio and co-workers [20]. Samples were thawed in 1X MAS buffer (70 mM sucrose, 220 mM mannitol, 5 mM KH_2_PO_4_, 5 mM MgCl_2_, 1 mM EGTA, 2 mM HEPES pH 7.4), finely minced with scissors, then homogenized with a glass Dounce homogenizer on ice as we have previously described [19]. The resulting homogenate was spun at 1000 *g* for 10 min at 4 °C. The supernatant was collected and immediately used for protein quantification by BCA assay (Thermo Scientific, 23225). Each well of the Seahorse microplate was loaded with the designated amount of homogenate protein (μg; Table 1), with each biological replicate measured in triplicate. To isolate CV-dependent activity, three baseline time points were first recorded. All samples were then treated with 4 μM rotenone (Complex I inhibitor) and 4 μM antimycin A (Complex III inhibitor), followed by three additional measurement time points. Complex V was subsequently activated by the addition of 20 mM ATP (Sigma A26209) together with 7.87 mM carbonyl cyanide p-trifluoromethoxyphenylhydrazone (FCCP; Enzo BML-CM120), an uncoupler of oxidative phosphorylation, and three more time points were collected. Finally, 10 μM oligomycin (Sigma 495455) was added to inhibit Complex V and distinguish non-mitochondrial acidification. Although the spun down supernatant we used is enriched for mitochondria, to prevent potential interference from V-type ATPases, we included 1 μM ouabain (Sigma 03125), a V-type–specific inhibitor, in the 1 × MAS medium during the assay. ATP synthase activity was calculated as the difference between the mean of three time points following ATP and FCCP injection and the mean of three time points following oligomycin injection.

**Table 1:** Assay parameters for ATP Synthase activity analysis across all tissues.

Tissue	Approximate Size	Protein used (μg/well)
Adipose - BAT	Both sides - entire	2
Adipose - gWAT	Both sides - entire	15
Adipose - iWAT	Both sides - entire	15
Brain - Cerebellum	Entire	6
Brain - Cortex	Both sides - 25 mg	6
Brain - Hippocampus	Both sides - entire	6
Brain - Hypothalamus	Entire	6
Eye	Both sides - entire	10
GI - Cecum	Entire	10
GI - Large intestine - Distal Colon	Entire	10
GI - Large intestine - Proximal Colon	Entire	10
GI - Small intestine - Duodenum	Entire	10
GI - Small intestine - Ileum	Entire	10
GI - Small intestine - Jejunum	Entire	10
Heart - atria	Both sides - entire	2
Heart - ventricle	Both sides - entire	2
Kidney - cortex	50–100 mg	6
Kidney - Medulla	50–100 mg	6
Liver	50–100 mg	8
Lung	Both sides - entire	10
Pancreas	Entire	8
Sex - Fallopian tubes	Entire	10
Sex - Testes	Single teste - entire	8
Skeletal muscle - Diaphragm	Entire	10
Skeletal muscle - Gastrocnemius	Both sides - entire	10
Skeletal muscle - Hamstring	Both sides - entire	8
Skeletal muscle - Plantaris	Both sides - entire	10
Skeletal muscle - Quadriceps	Both sides - entire	10
Skeletal muscle - Soleus	Both sides - entire	8
Skeletal muscle - Tongue	Entire	8
Skin	100–200 mg	10
Spleen	Entire	8
Stomach	Entire	8

### 2.4 Quantification of mitochondrial content

Mitochondrial content of homogenates was quantified with MitoTracker Deep Red FM (MTDR, Invitrogen, M22426) staining as previously described [19]. Briefly, lysates were incubated with MTDR (1 μM) for 10 min at 37 °C, then centrifuged at 2000 *g* for 5 min at 4 °C. The supernatant was carefully removed and replaced with 1X MAS solution, and fluorescence was read with excitation and emission wavelengths of 625 nm and 670 nm, respectively. All measurements were read with a BioTek Synergy HTX Multimode Plate Reader (Agilent). All samples from a single tissue-type were measured on the same plate, in duplicate, with an equal gain setting of 90 to ensure comparability across samples. To minimize non-specific background signal contribution, control wells were loaded with MTDR + 1X MAS and subtracted from all sample values.

### 2.5 ATP synthase related gene expression in mouse and human based on age

Mouse [15] and human [21] bulk RNA-seq data were obtained from GEO using the accession GSE132040 or GTEx V8 downloads portal, respectively. Nineteen genes directly associated with ATP synthase activity were extracted for downstream analysis (Table 2). Mouse and human orthologues were obtained from the Mouse Genome Informatics (MGI) database. For the mouse transcriptomic data [15], tissues were collected from males (*n* = 4) and virgin females (*n* = 2) at 1, 3, 6, 9, 12, 15, 18, 21,24 and 27 months of age. For this study, mouse tissue samples ≤15 months were classified as young and those >15 months as old. Mouse tissue groups included in the analysis were: white blood cells (WBC), spleen, bone marrow (Marrow), bone, brown adipose tissue (BAT), gonadal white adipose tissue (gWAT), inguinal/subcutaneous white adipose tissue (iWAT), duodenum, pancreas, dorsal skin, tibialis anterior skeletal muscle (Muscle), heart, kidney, lung, liver, and brain. For the human transcriptomic data from GTEx (v.8) [21], sample size consists of 259 males and 186 females. Human tissues included in our analysis: subcutaneous adipose (SubQ adipose), visceral adipose, adrenal gland, aorta, coronary artery, tibial artery, amygdala, anterior cingulate cortex, caudate, cerebellum, cerebellar hemisphere (cerebellar hemi), cortex, frontal cortex, hippocampus, hypothalamus, nucleus accumbens, putamen, spinal cord, substantia nigra, fibroblasts, lymphoblasts, sigmoid colon, transverse colon, esophageal gastroesophageal junction (esoph. GE junction), esophageal mucosa, esophageal muscle, heart atrium, heart ventricle, kidney cortex, liver, lung, salivary gland, skeletal muscle, tibial nerve, pancreas, pituitary, non-exposed skin, exposed skin, ileum, spleen, stomach, thyroid, and blood. Male reproductive tissues included prostate and testis, while female reproductive tissues included ovary, uterus, and vagina. Expression matrices were generated corresponding to individuals as rows and gene_tissue as columns where columns detected at a TPM >1 in at least 30% of individuals were retained. Genes used in Table 2 were then compared between older (over 50 years of age in humans and 1.5 years of age in mice) and younger individuals. Empirical *p*-values were calculated based on two-way t-tests where FDR adjustments were made based on the number of tissues being compared using the qvalue(.) package in R.

**Table 2:** Human and mouse ATP synthase related genes used in expression analysis.

Human Gene	Mouse Ortholog	Complex/Subunit	Function/Annotation
MT-ATP6	Mt-Atp6	Core subunit (F_0_)	Encodes ATP synthase subunit a
MT-ATP8	Mt-Atp8	Core subunit (F_0_)	Encodes ATP synthase subunit A6L
ATP5F1A	Atp5f1a	F_1_ subunit	Alpha subunit, catalytic core
ATP5F1B	Atp5f1b	F_1_ subunit	Beta subunit, catalytic core
ATP5F1C	Atp5f1c	F_1_ subunit	Gamma subunit, catalytic core
ATP5F1D	Atp5f1d	F_1_ subunit	Delta subunit, catalytic core
ATP5F1E	Atp5f1e	F_1_ subunit	Epsilon subunit, catalytic core
ATP5MC1	Atp5mc1	F_0_ subunit	Membrane subunit c, isoform 1
ATP5MC2	Atp5mc2	F_0_ subunit	Membrane subunit c, isoform 2
ATP5MC3	Atp5mc3	F_0_ subunit	Membrane subunit c, isoform 3
ATP5ME	Atp5me	F_0_ subunit	Epsilon subunit of F_0_
ATP5MF	Atp5mf	F_0_ subunit	Subunit f of F_0_
ATP5MG	Atp5mg	F_0_ subunit	Subunit g of F_0_
ATP5PB	Atp5pb	F_0_ subunit	Subunit b of F_0_
ATP5PD	Atp5pd	F_0_ subunit	Subunit d of F_0_
ATP5PO	Atp5po	F_0_ subunit	Oligomycin sensitivity
ATPAF1	Atpaf1	Assembly factor	Essential for F_1_ complex assembly
ATPAF2	Atpaf2	Assembly factor	Essential for F_1_ complex assembly
TMEM70	Tmem70	Assembly factor	Involved in F_0_–F_1_ assembly

## 3 Results

### 3.1 Overview of Pan tissue ATP synthase activity, workflow, and analysis pipeline

To assay ATP synthase (Complex V, CV) hydrolytic activity across age and sex, we collected 32 tissues from young (10 weeks) and old (80 weeks) male and female mice (*n* = 10 per group). The tissues collected included multiple brain regions (hippocampus, cortex, cerebellum, and hypothalamus), sections of the gastrointestinal tract (stomach, duodenum, ileum, jejunum, cecum, proximal colon, and distal colon), major adipose depots (gonadal, inguinal, brown), diverse skeletal muscle groups (tongue, diaphragm, quadriceps complex, hamstrings, gastrocnemius, plantaris, and soleus), reproductive organs (testis and fallopian tubes), as well as liver, pancreas, heart atrium and ventricle, spleen, kidney cortex and medulla, eye, and skin (Figure 1A). Our goal was to generate a comprehensive, systems-level atlas of ATP synthase activity across age and sex.

**Supplementary Figure 1:**
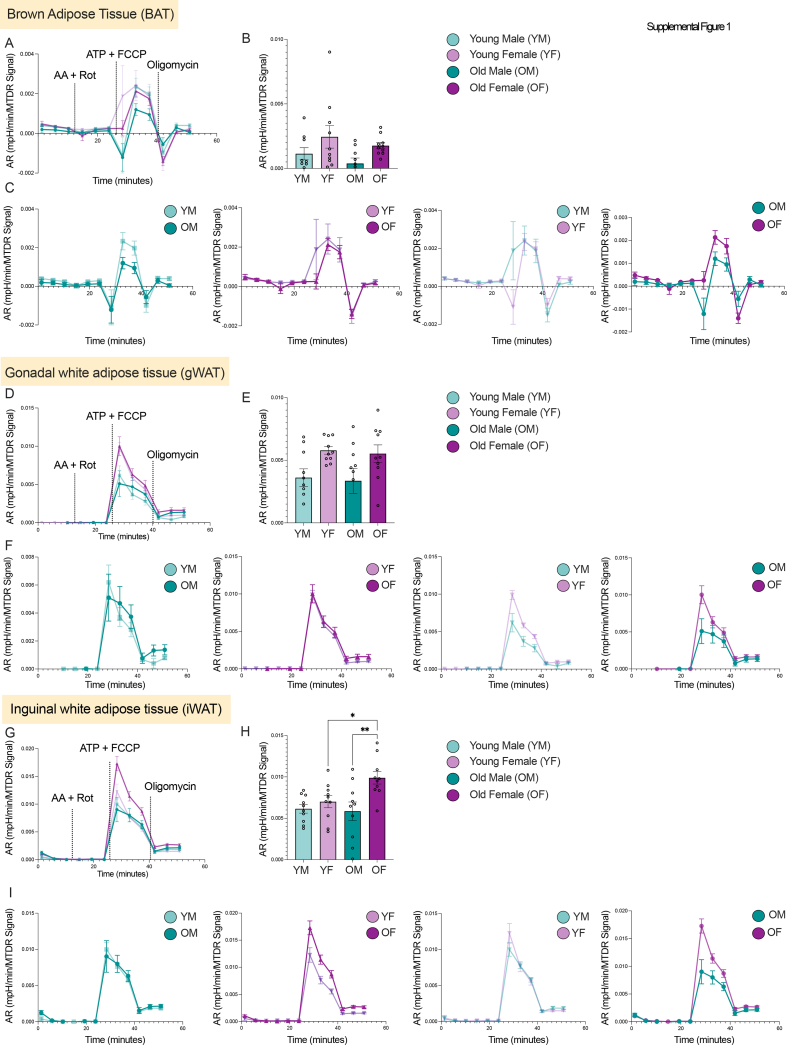
ATP synthase hydrolytic activity in brown adipose tissue (BAT), gonadal white adipose tissue (gWAT), and inguinal white adipose tissue (iWAT). (A) ATP synthase hydrolytic activity traces of BAT from all groups (young male, young female, old male, old female; n = 10 per group), normalized to mitochondrial content as determined by MTDR. Sequential injections are indicated: (i) basal reads; (ii) Antimycin A (AA, Complex III inhibitor) and Rotenone (Rot, Complex I inhibitor); (iii) ATP (Complex V substrate) and FCCP (ETC uncoupler); and (iv) Oligomycin (ATP synthase inhibitor). (B) Average oligomycin-sensitive ATP synthase hydrolytic activity in BAT, normalized to mitochondrial content. Each dot represents the mean of three technical replicates measured independently across three runs. (C) Group-wise comparisons of BAT activity, normalized to mitochondrial content. From left to right: 1) young male vs. old male; 2) young female vs. old female; 3) young male vs. young female; and 4) old male vs. old female. (D–F) ATP synthase hydrolytic activity in gWAT. (D) Traces from all groups (n = 10 per group). (E) Average oligomycin-sensitive activity, normalized to mitochondrial content; each dot represents three independent technical replicates. (F) Group comparisons: young vs. old (male, female) and male vs. female (young, old). (G–I) ATP synthase hydrolytic activity in iWAT. (G) Traces from all groups (n = 10 per group). (H) Average oligomycin-sensitive activity, normalized to mitochondrial content; each dot represents three independent technical replicates. (I) Group comparisons: young vs. old (male, female) and male vs. female (young, old).

**Supplementary Figure 2:**
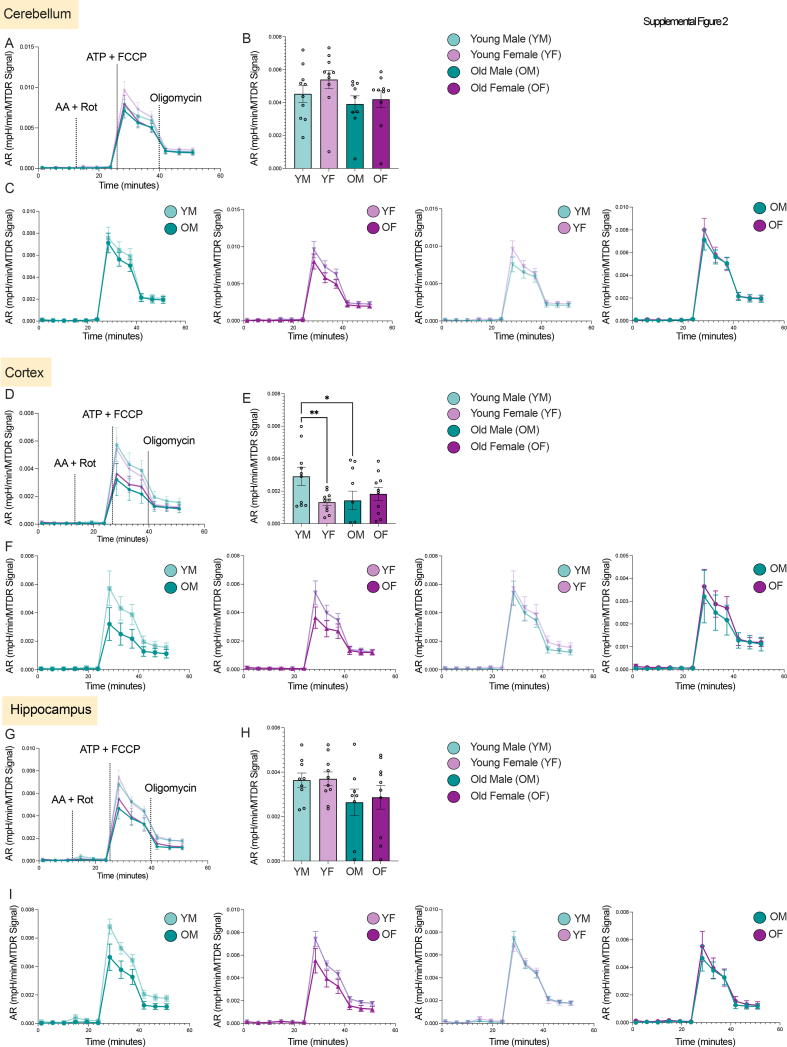
ATP synthase hydrolytic activity in cerebellum, cortex, and hippocampus. (A) ATP synthase hydrolytic activity traces of cerebellum from all groups (young male, young female, old male, old female; n = 10 per group), normalized to mitochondrial content as determined by MTDR. Sequential injections are indicated: (i) basal reads; (ii) Antimycin A (AA, Complex III inhibitor) and Rotenone (Rot, Complex I inhibitor); (iii) ATP (Complex V substrate) and FCCP (ETC uncoupler); and (iv) Oligomycin (ATP synthase inhibitor). (B) Average oligomycin-sensitive ATP synthase hydrolytic activity in cerebellum, normalized to mitochondrial content. Each dot represents the mean of three technical replicates measured independently across three runs. (C) Group-wise comparisons of cerebellum activity, normalized to mitochondrial content. From left to right: 1) young male vs. old male; 2) young female vs. old female; 3) young male vs. young female; and 4) old male vs. old female. (D–F) ATP synthase hydrolytic activity in cortex. (D) Traces from all groups (n = 10 per group). (E) Average oligomycin-sensitive activity, normalized to mitochondrial content; each dot represents three independent technical replicates. (F) Group comparisons: young vs. old (male, female) and male vs. female (young, old). (G–I) ATP synthase hydrolytic activity in hippocampus. (G) Traces from all groups (n = 10 per group). (H) Average oligomycin-sensitive activity, normalized to mitochondrial content; each dot represents three independent technical replicates. (I) Group comparisons: young vs. old (male, female) and male vs. female (young, old).

**Supplementary Figure 3:**
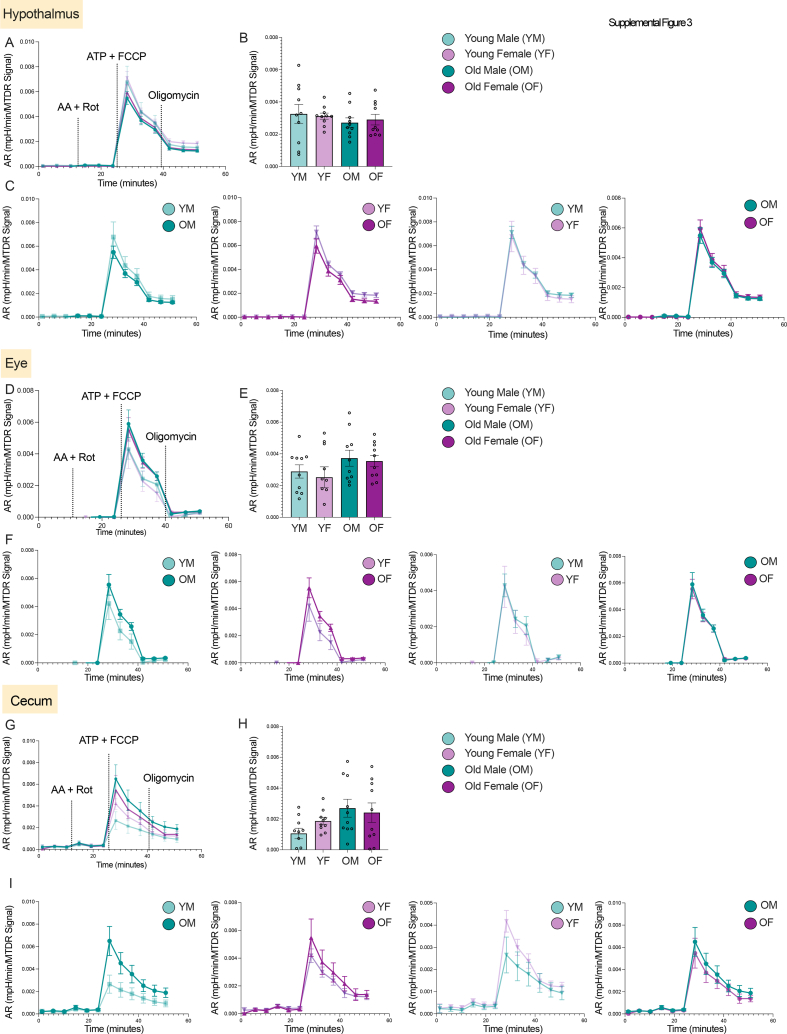
ATP synthase hydrolytic activity in hypothalamus, eye, and cecum. (A) ATP synthase hydrolytic activity traces of hypothalamus from all groups (young male, young female, old male, old female; n = 10 per group), normalized to mitochondrial content as determined by MTDR. Sequential injections are indicated: (i) basal reads; (ii) Antimycin A (AA, Complex III inhibitor) and Rotenone (Rot, Complex I inhibitor); (iii) ATP (Complex V substrate) and FCCP (ETC uncoupler); and (iv) Oligomycin (ATP synthase inhibitor). (B) Average oligomycin-sensitive ATP synthase hydrolytic activity in hypothalamus, normalized to mitochondrial content. Each dot represents the mean of three technical replicates measured independently across three runs. (C) Group-wise comparisons of hypothalamus activity, normalized to mitochondrial content. From left to right: 1) young male vs. old male; 2) young female vs. old female; 3) young male vs. young female; and 4) old male vs. old female. (D–F) ATP synthase hydrolytic activity in eye. (D) Traces from all groups (n = 10 per group). (E) Average oligomycin-sensitive activity, normalized to mitochondrial content; each dot represents three independent technical replicates. (F) Group comparisons: young vs. old (male, female) and male vs. female (young, old). (G–I) ATP synthase hydrolytic activity in cecum. (G) Traces from all groups (n = 10 per group). (H) Average oligomycin-sensitive activity, normalized to mitochondrial content; each dot represents three independent technical replicates. (I) Group comparisons: young vs. old (male, female) and male vs. female (young, old).

**Supplementary Figure 4:**
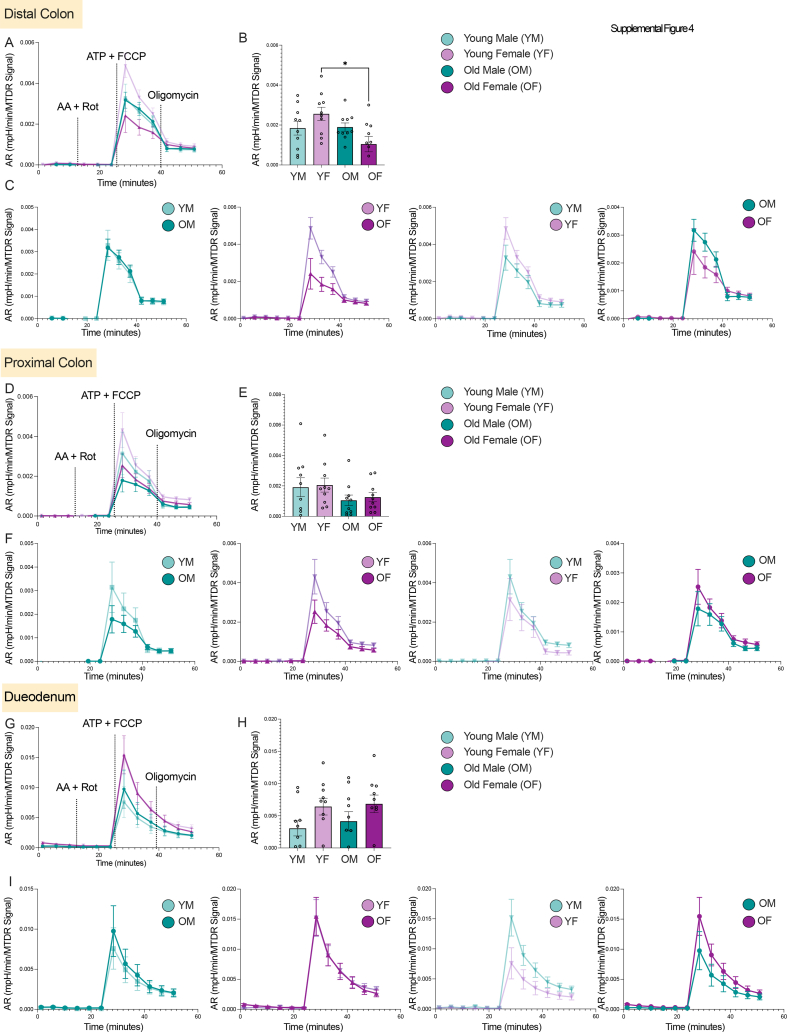
ATP synthase hydrolytic activity in distal colon, proximal colon, and duodenum. (A) ATP synthase hydrolytic activity traces of distal colon from all groups (young male, young female, old male, old female; n = 10 per group), normalized to mitochondrial content as determined by MTDR. Sequential injections are indicated: (i) basal reads; (ii) Antimycin A (AA, Complex III inhibitor) and Rotenone (Rot, Complex I inhibitor); (iii) ATP (Complex V substrate) and FCCP (ETC uncoupler); and (iv) Oligomycin (ATP synthase inhibitor). (B) Average oligomycin-sensitive ATP synthase hydrolytic activity in distal colon, normalized to mitochondrial content. Each dot represents the mean of three technical replicates measured independently across three runs. (C) Group-wise comparisons of distal colon activity, normalized to mitochondrial content. From left to right: 1) young male vs. old male; 2) young female vs. old female; 3) young male vs. young female; and 4) old male vs. old female. (D–F) ATP synthase hydrolytic activity in proximal colon. (D) Traces from all groups (n = 10 per group). (E) Average oligomycin-sensitive activity, normalized to mitochondrial content; each dot represents three independent technical replicates. (F) Group comparisons: young vs. old (male, female) and male vs. female (young, old). (G–I) ATP synthase hydrolytic activity in duodenum. (G) Traces from all groups (n = 10 per group). (H) Average oligomycin-sensitive activity, normalized to mitochondrial content; each dot represents three independent technical replicates. (I) Group comparisons: young vs. old (male, female) and male vs. female (young, old).

**Supplementary Figure 5:**
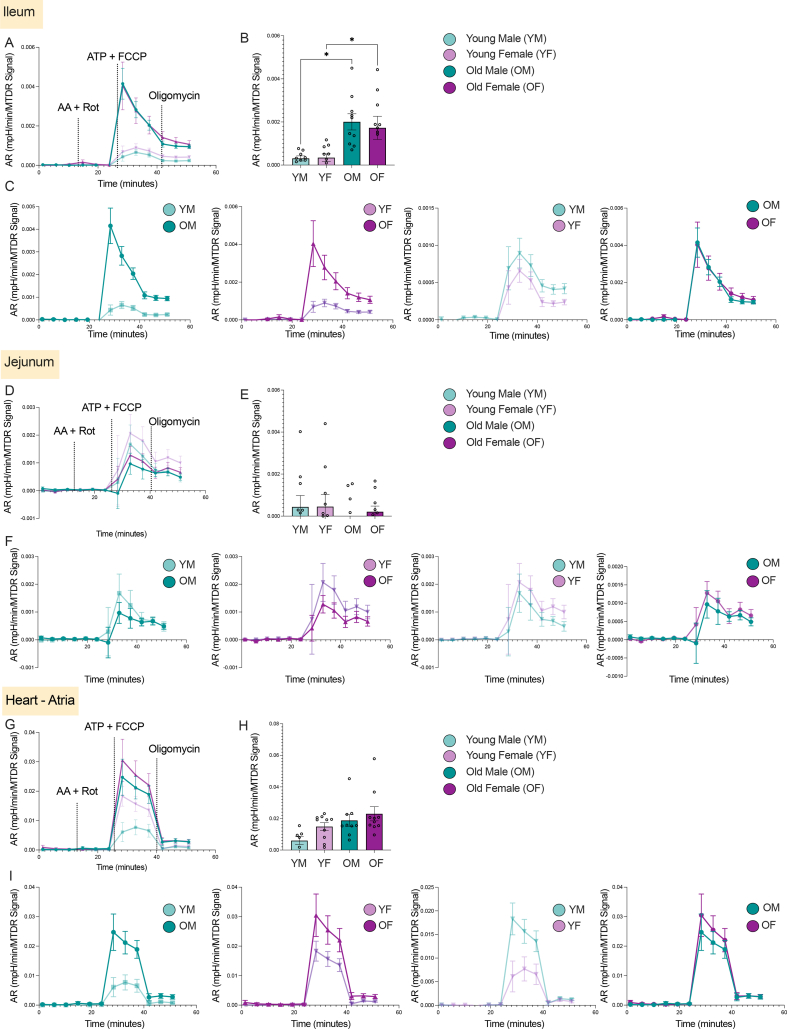
ATP synthase hydrolytic activity in ileum, jejunum, and heart atrium. (A) ATP synthase hydrolytic activity traces of ileum from all groups (young male, young female, old male, old female; n = 10 per group), normalized to mitochondrial content as determined by MTDR. Sequential injections are indicated: (i) basal reads; (ii) Antimycin A (AA, Complex III inhibitor) and Rotenone (Rot, Complex I inhibitor); (iii) ATP (Complex V substrate) and FCCP (ETC uncoupler); and (iv) Oligomycin (ATP synthase inhibitor). (B) Average oligomycin-sensitive ATP synthase hydrolytic activity in ileum, normalized to mitochondrial content. Each dot represents the mean of three technical replicates measured independently across three runs. (C) Group-wise comparisons of ileum activity, normalized to mitochondrial content. From left to right: 1) young male vs. old male; 2) young female vs. old female; 3) young male vs. young female; and 4) old male vs. old female. (D–F) ATP synthase hydrolytic activity in jejunum. (D) Traces from all groups (n = 10 per group). (E) Average oligomycin-sensitive activity, normalized to mitochondrial content; each dot represents three independent technical replicates. (F) Group comparisons: young vs. old (male, female) and male vs. female (young, old). (G–I) ATP synthase hydrolytic activity in heart atrium. (G) Traces from all groups (n = 10 per group). (H) Average oligomycin-sensitive activity, normalized to mitochondrial content; each dot represents three independent technical replicates. (I) Group comparisons: young vs. old (male, female) and male vs. female (young, old).

**Supplementary Figure 6:**
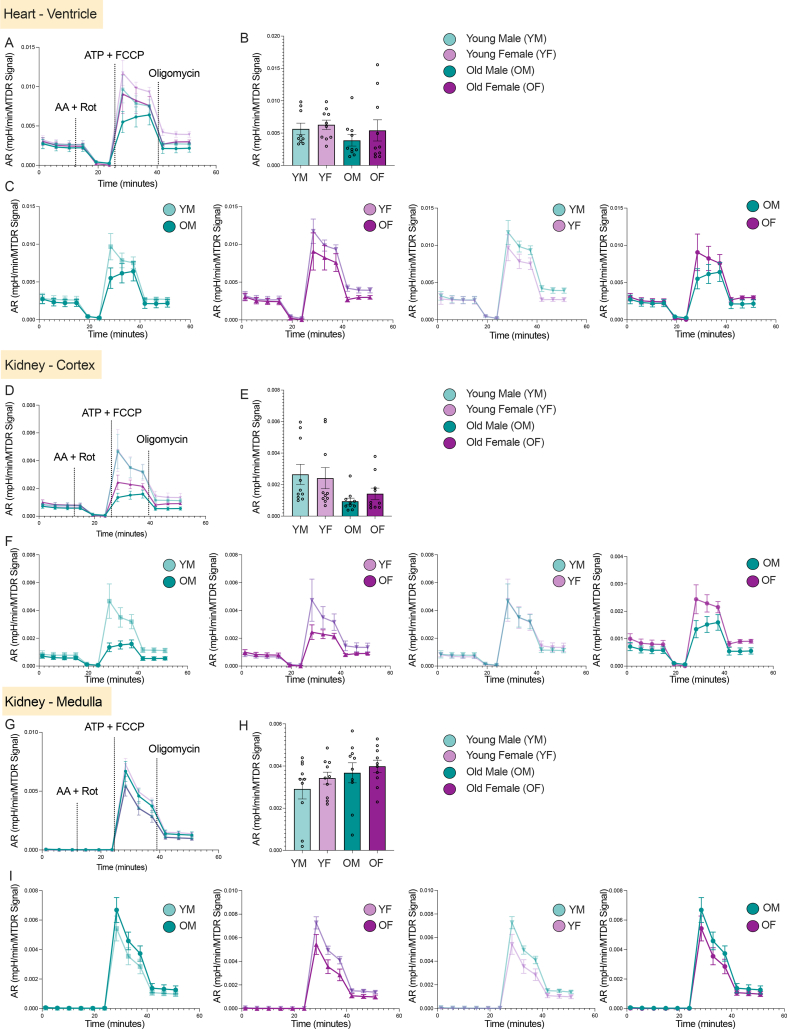
ATP synthase hydrolytic activity in heart ventricles, kidney cortex, and kidney medulla. (A) ATP synthase hydrolytic activity traces of heart ventricle from all groups (young male, young female, old male, old female; n = 10 per group), normalized to mitochondrial content as determined by MTDR. Sequential injections are indicated: (i) basal reads; (ii) Antimycin A (AA, Complex III inhibitor) and Rotenone (Rot, Complex I inhibitor); (iii) ATP (Complex V substrate) and FCCP (ETC uncoupler); and (iv) Oligomycin (ATP synthase inhibitor). (B) Average oligomycin-sensitive ATP synthase hydrolytic activity in heart ventricles, normalized to mitochondrial content. Each dot represents the mean of three technical replicates measured independently across three runs. (C) Group-wise comparisons of heart ventricle activity, normalized to mitochondrial content. From left to right: 1) young male vs. old male; 2) young female vs. old female; 3) young male vs. young female; and 4) old male vs. old female. (D–F) ATP synthase hydrolytic activity in kidney cortex. (D) Traces from all groups (n = 10 per group). (E) Average oligomycin-sensitive activity, normalized to mitochondrial content; each dot represents three independent technical replicates. (F) Group comparisons: young vs. old (male, female) and male vs. female (young, old). (G–I) ATP synthase hydrolytic activity in kidney medulla. (G) Traces from all groups (n = 10 per group). (H) Average oligomycin-sensitive activity, normalized to mitochondrial content; each dot represents three independent technical replicates. (I) Group comparisons: young vs. old (male, female) and male vs. female (young, old).

**Supplementary Figure 7:**
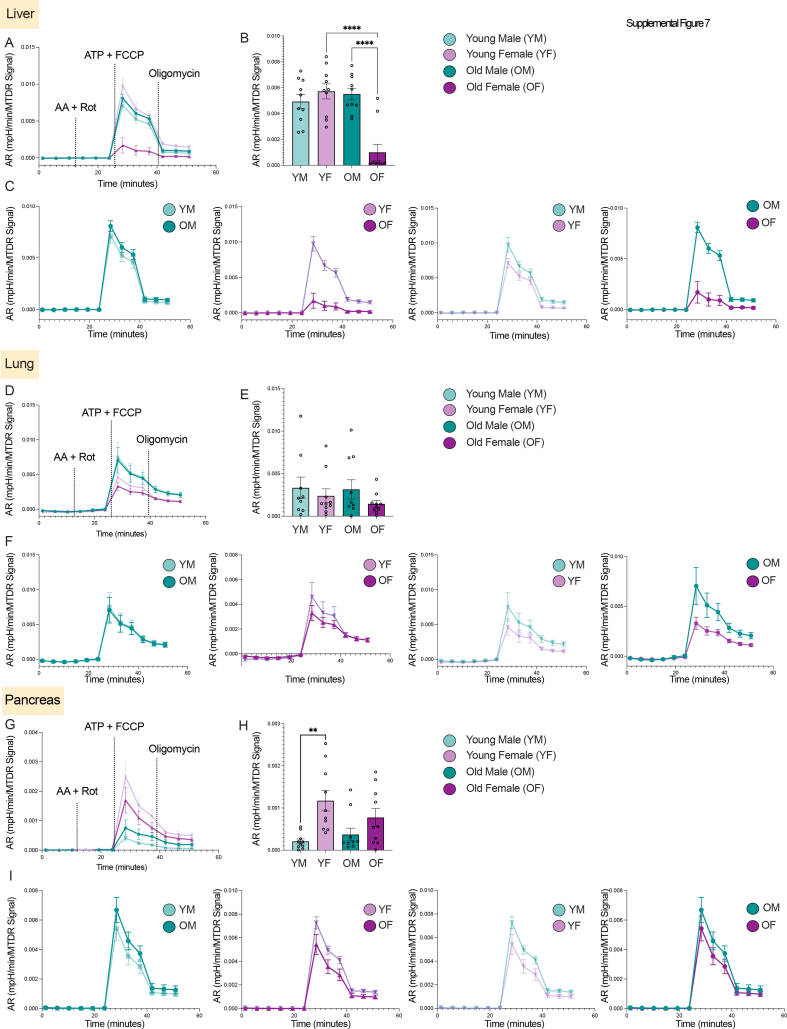
ATP synthase hydrolytic activity in liver, lung, and pancreas (A) ATP synthase hydrolytic activity traces of Liver from all groups (young male, young female, old male, old female; n = 10 per group), normalized to mitochondrial content as determined by MTDR. Sequential injections are indicated: (i) basal reads; (ii) Antimycin A (AA, Complex III inhibitor) and Rotenone (Rot, Complex I inhibitor); (iii) ATP (Complex V substrate) and FCCP (ETC uncoupler); and (iv) Oligomycin (ATP synthase inhibitor). (B) Average oligomycin-sensitive ATP synthase hydrolytic activity in liver, normalized to mitochondrial content. Each dot represents the mean of three technical replicates measured independently across three runs. (C) Group-wise comparisons of liver activity, normalized to mitochondrial content. From left to right: 1) young male vs. old male; 2) young female vs. old female; 3) young male vs. young female; and 4) old male vs. old female. (D–F) ATP synthase hydrolytic activity in lung. (D) Traces from all groups (n = 10 per group). (E) Average oligomycin-sensitive activity, normalized to mitochondrial content; each dot represents three independent technical replicates. (F) Group comparisons: young vs. old (male, female) and male vs. female (young, old). (G–I) ATP synthase hydrolytic activity in pancreas. (G) Traces from all groups (n = 10 per group). (H) Average oligomycin-sensitive activity, normalized to mitochondrial content; each dot represents three independent technical replicates. (I) Group comparisons: young vs. old (male, female) and male vs. female (young, old).

**Supplementary Figure 8:**
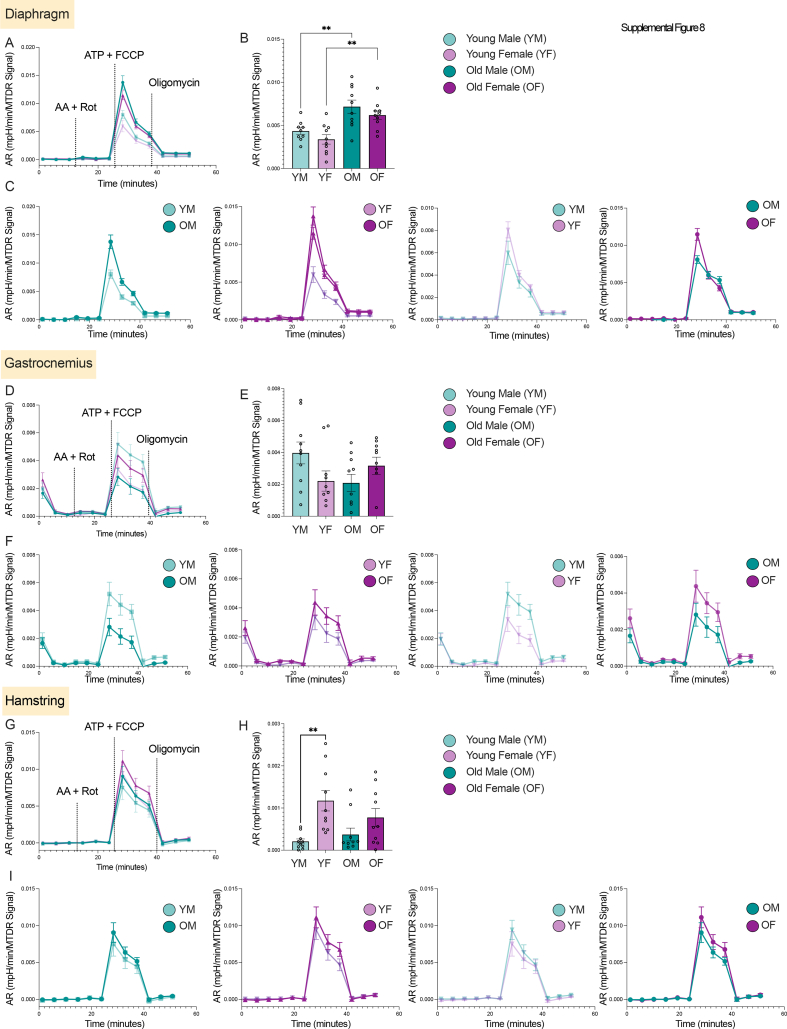
ATP synthase hydrolytic activity in diaphragm, gastrocnemius, and hamstring. (A) ATP synthase hydrolytic activity traces of diaphragm from all groups (young male, young female, old male, old female; n = 10 per group), normalized to mitochondrial content as determined by MTDR. Sequential injections are indicated: (i) basal reads; (ii) Antimycin A (AA, Complex III inhibitor) and Rotenone (Rot, Complex I inhibitor); (iii) ATP (Complex V substrate) and FCCP (ETC uncoupler); and (iv) Oligomycin (ATP synthase inhibitor). (B) Average oligomycin-sensitive ATP synthase hydrolytic activity in diaphragm, normalized to mitochondrial content. Each dot represents the mean of three technical replicates measured independently across three runs. (C) Group-wise comparisons of diaphragm activity, normalized to mitochondrial content. From left to right: 1) young male vs. old male; 2) young female vs. old female; 3) young male vs. young female; and 4) old male vs. old female. (D–F) ATP synthase hydrolytic activity in gastrocnemius. (D) Traces from all groups (n = 10 per group). (E) Average oligomycin-sensitive activity, normalized to mitochondrial content; each dot represents three independent technical replicates. (F) Group comparisons: young vs. old (male, female) and male vs. female (young, old). (G–I) ATP synthase hydrolytic activity in hamstring. (G) Traces from all groups (n = 10 per group). (H) Average oligomycin-sensitive activity, normalized to mitochondrial content; each dot represents three independent technical replicates. (I) Group comparisons: young vs. old (male, female) and male vs. female (young, old).

**Supplementary Figure 9:**
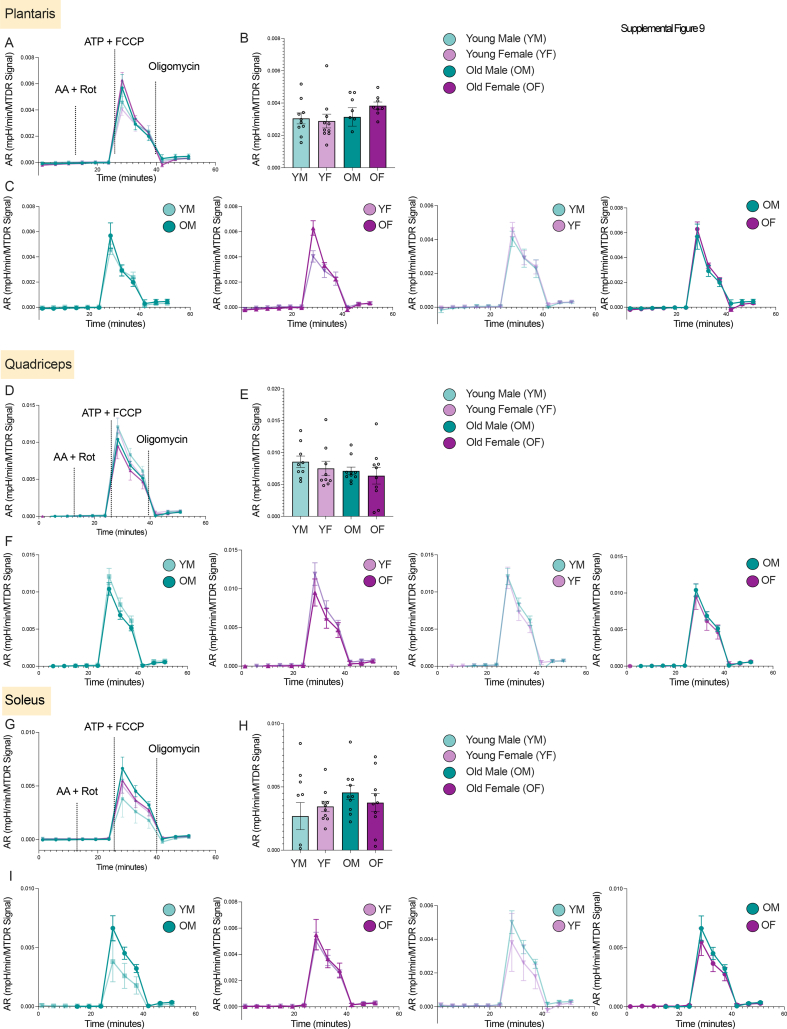
ATP synthase hydrolytic activity in plantaris, quadriceps, and soleus. (A) ATP synthase hydrolytic activity traces of plantaris from all groups (young male, young female, old male, old female; n = 10 per group), normalized to mitochondrial content as determined by MTDR. Sequential injections are indicated: (i) basal reads; (ii) Antimycin A (AA, Complex III inhibitor) and Rotenone (Rot, Complex I inhibitor); (iii) ATP (Complex V substrate) and FCCP (ETC uncoupler); and (iv) Oligomycin (ATP synthase inhibitor).(B) Average oligomycin-sensitive ATP synthase hydrolytic activity in plantaris, normalized to mitochondrial content. Each dot represents the mean of three technical replicates measured independently across three runs. (C) Group-wise comparisons of plantaris activity, normalized to mitochondrial content. From left to right: 1) young male vs. old male; 2) young female vs. old female; 3) young male vs. young female; and 4) old male vs. old female. (D–F) ATP synthase hydrolytic activity in quadriceps. (D) Traces from all groups (n = 10 per group). (E) Average oligomycin-sensitive activity, normalized to mitochondrial content; each dot represents three independent technical replicates. (F) Group comparisons: young vs. old (male, female) and male vs. female (young, old). (G–I) ATP synthase hydrolytic activity in soleus. (G) Traces from all groups (n = 10 per group). (H) Average oligomycin-sensitive activity, normalized to mitochondrial content; each dot represents three independent technical replicates. (I) Group comparisons: young vs. old (male, female) and male vs. female (young, old).

**Supplementary Figure 10:**
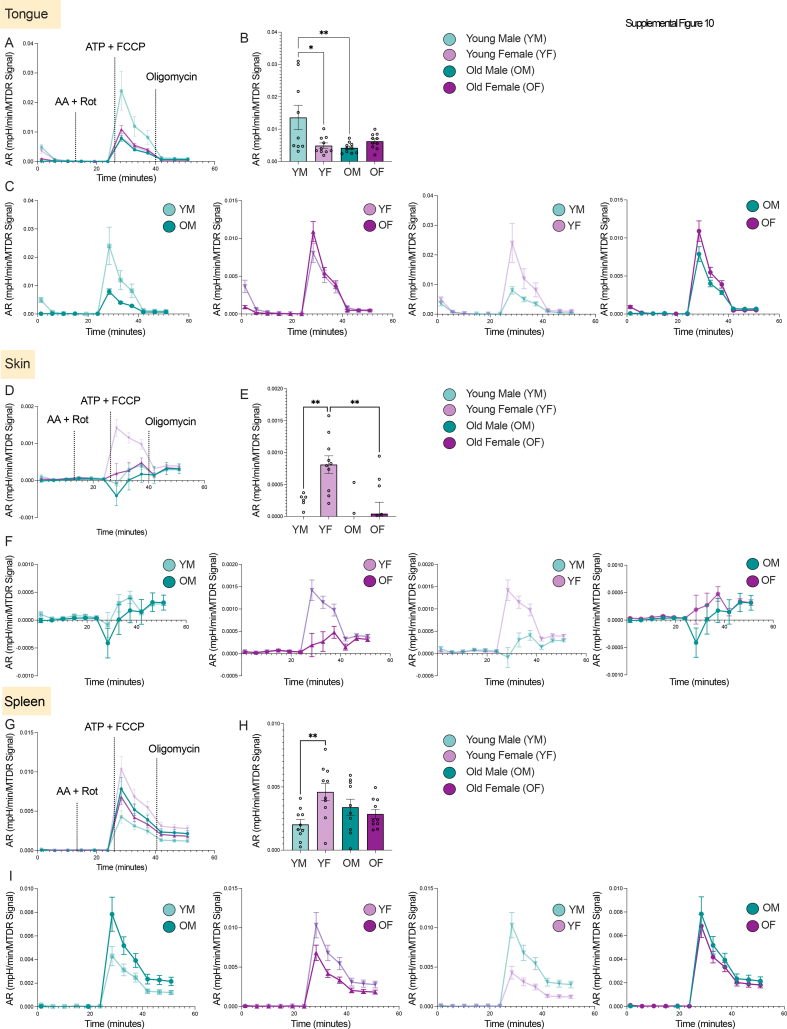
ATP synthase hydrolytic activity in tongue, skin, and spleen. (A) ATP synthase hydrolytic activity traces of tongue from all groups (young male, young female, old male, old female; n = 10 per group), normalized to mitochondrial content as determined by MTDR. Sequential injections are indicated: (i) basal reads; (ii) Antimycin A (AA, Complex III inhibitor) and Rotenone (Rot, Complex I inhibitor); (iii) ATP (Complex V substrate) and FCCP (ETC uncoupler); and (iv) Oligomycin (ATP synthase inhibitor). (B) Average oligomycin-sensitive ATP synthase hydrolytic activity in tongue, normalized to mitochondrial content. Each dot represents the mean of three technical replicates measured independently across three runs. (C) Group-wise comparisons of tongue activity, normalized to mitochondrial content. From left to right: 1) young male vs. old male; 2) young female vs. old female; 3) young male vs. young female; and 4) old male vs. old female. (D–F) ATP synthase hydrolytic activity in skin. (D) Traces from all groups (n = 10 per group). (E) Average oligomycin-sensitive activity, normalized to mitochondrial content; each dot represents three independent technical replicates. (F) Group comparisons: young vs. old (male, female) and male vs. female (young, old). (G–I) ATP synthase hydrolytic activity in spleen. (G) Traces from all groups (n = 10 per group) Traces from all groups (n = 10 per group). (H) Average oligomycin-sensitive activity, normalized to mitochondrial content; each dot represents three independent technical replicates. (I) Group comparisons: young vs. old (male, female) and male vs. female (young, old).

**Supplementary Figure 11:**
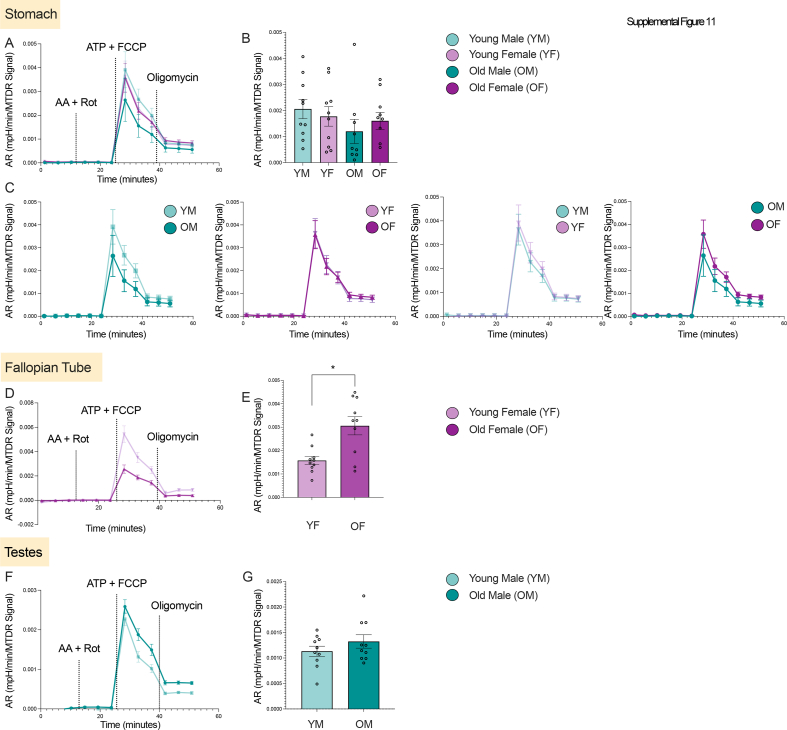
ATP synthase hydrolytic activity of stomach, fallopian tube, and testes. (A) ATP synthase hydrolytic activity of stomach for all groups (young male, young female, old male, old female; n = 10 per group), normalized to mitochondrial content as determined by MTDR. Sequential injections are indicated: i) basal reads; ii) Antimycin A (AA, Complex III inhibitor) and Rotenone (Rot, Complex I inhibitor); iii) ATP (Complex V substrate) and FCCP (ETC uncoupler); and iv) Oligomycin (ATP synthase inhibitor). (B) Average values of oligomycin sensitive ATP synthase hydrolytic activity in stomach normalized to mitochondrial content. Each dot represents the average of three technical replicates measured at three separate times. (C) ATP Synthase hydrolytic activity traces normalized to mitochondrial content. From left to right 1) young male vs old male, 2) Young female vs old female, 3) young male vs. young female and 4) old male vs. old female. (D) ATP synthase hydrolytic activity of fallopian tubes for young and old female normalized to mitochondrial content. (E) Average values of oligomycin sensitive ATP synthase hydrolytic activity in fallopian tubes normalized to mitochondrial content. Each dot represents the average of three technical replicates measured at three separate times. (F) ATP synthase hydrolytic activity of testes for young and old male normalized to mitochondrial content. (G) Average values of oligomycin sensitive ATP synthase hydrolytic activity in testes normalized to mitochondrial content. Each dot represents the average of three technical replicates measured at three separate times.

Our standardized workflow began with thawing frozen tissue samples, followed by mincing and homogenization. Homogenates were centrifuged and the supernatant was collected for protein quantification. Maximal ATP synthase hydrolytic capacity was then assayed using a Seahorse XFe96 Analyzer with a stepwise protocol (Figure 1B). The assay consisted of four sequential steps: (i) baseline acidification rates were obtained, (ii) rotenone (Rot, Complex I inhibitor) and antimycin A (AA, Complex III inhibitor) were added to inhibit electron transport chain, thereby preventing any forward rotation of CV, (iii) ATP together with FCCP (to dissipate any remaining membrane potential) was added to drive ATP hydrolysis with the concomitant production of H^+^ which acidified the medium over time, leading to a change in pH as indicated by the acidification rate (AR); (iv) oligomycin was added to inhibit Complex V to indicate the proportion of acidification attributable to CV. For our assay, we included ouabain, a V-type ATPase inhibitor, in the medium to prevent any potential interference from non-mitochondrial ATPases. This analysis pipeline allowed direct measurement of ATP synthase hydrolytic capacity, with outputs expressed as changes in acidification rate (AR) linked to ATP hydrolysis (Figure 1B; Supplementary Table 1 and Supplementary Figs. 1–11). Together, the assay provides a standardized procedure and platform for determining ATP synthase function across tissues, sex, and age, offering a rich dataset that complements the mitochondrial respiration atlas we previously generated [19].

### 3.2 ATP synthase activity across age and sex

**Figure 2:**
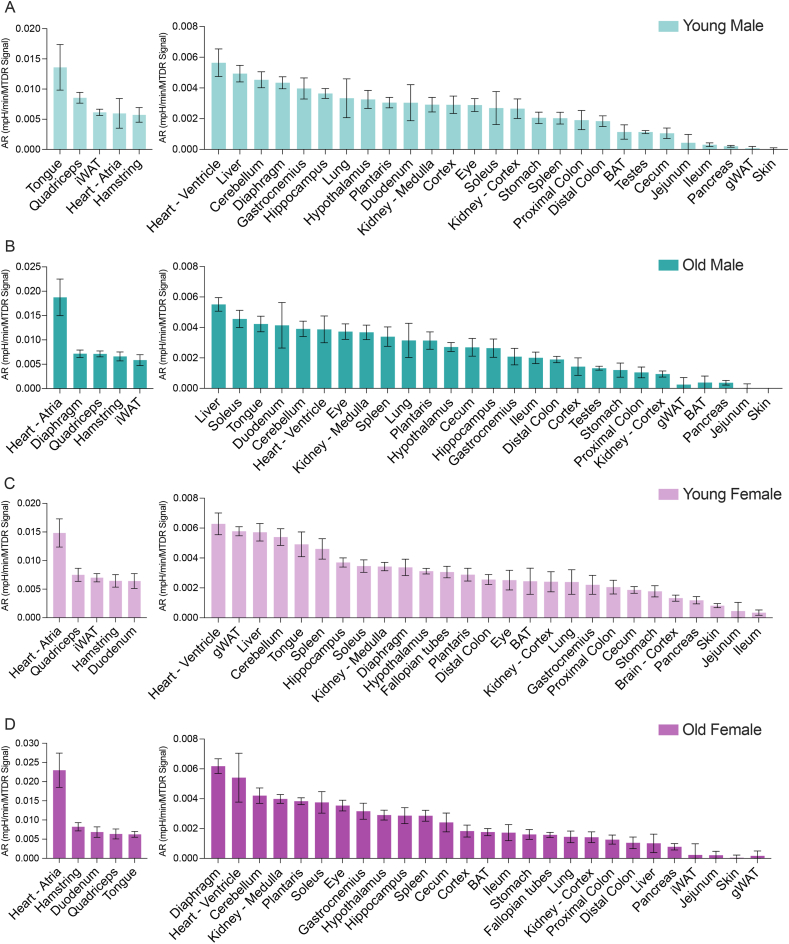
**ATP synthase activity across tissues in young and old male and female mice**. (A-B) ATP synthase activity in young (A) and old (B) male mice. (C-D) ATP synthase activity in young (C) and old (D) female mice. All ATP synthase hydrolytic rates, reflected by the acidification rate (AR), are normalized to mitochondrial content, as determined by MTDR. Data is presented as the mean with standard error, arranged from highest (left) to lowest (right) values. Young refers to 10-week-old mice, and old refers to 80-week-old mice. Abbreviations: BAT, brown adipose tissue; gWAT, gonadal white adipose tissue; iWAT, inguinal white adipose tissue.

Ranking young male tissues by ATP synthase hydrolytic activity showed that the tongue, quadriceps, inguinal white adipose tissue (iWAT), heart atrium, and hamstrings have the highest activity (Figure 2A). Most striated muscle groups (heart ventricle, diaphragm, gastrocnemius, plantaris), brain regions (cerebellum, hippocampus, and hypothalamus), as well as liver, lung, and kidney had intermediate CV activity. In contrast, gastrointestinal tissues such as the distal colon, ileum, and jejunum, as well as pancreas, skin, and gonadal white adipose tissue (gWAT) displayed low CV activity. In old males, CV activity declined across many contractile tissues, although the heart ventricle, iWAT, quadriceps, and hamstrings maintained stable CV activity (Figure 2B). Several brain regions in males also had stable CV activity with age. Skin, brown adipose tissue (BAT), gWAT, pancreas, and jejunum displayed low CV activity regardless of age.

The distribution of CV activity in young females differed from that in males. High activity was detected not only in the heart atrium, quadriceps, and hamstrings, but also in the duodenum, gWAT, and spleen (Figure 2C). Fallopian tube and BAT also displayed appreciable activity. Like young males, young females also had low CV activity in the skin, jejunum, and ileum. Ranking old female tissues by CV activity revealed a different aging trajectory compared to males (Figure 2D). Direct comparisons highlighted similarities and differences between males and females. For example, both sexes showed consistently high CV activity in the heart atrium, quadriceps, and hamstrings regardless of age. Aged females had high CV activity in the duodenum, tongue, diaphragm, and plantaris, while aged males had high CV activity in iWAT, liver, and soleus. Tissue types of high similarity were evident, such as different brain regions and the kidney cortex versus medulla, while others displayed striking differences, including heart atrium versus ventricle, distinct skeletal muscle groups, white adipose depots, and different segments of the gastrointestinal tract. Together, these data indicate wide variations in ATP synthase activity across tissues and age in both sexes.

### 3.3 Age-dependent changes in ATP synthase activity

**Figure 3:**
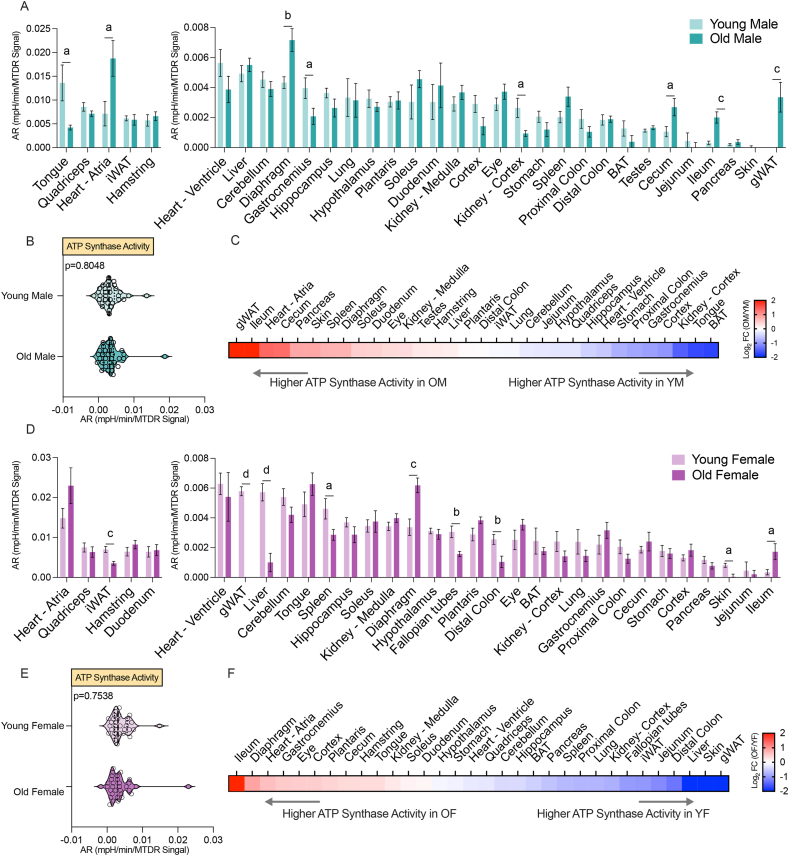
**Age-dependent changes in ATP synthase activity in male and female tissues**. (A) Comparison of ATP synthase activity, reflected by the acidification rate (AR), in young and old male tissues. Data is presented as the mean with standard error, arranged from highest (left) to lowest (right) values, and normalized to mitochondrial content (MTDR). (B) Systems-level comparison of ATP synthase activity in all 32 tissues between young and old males. (C) Heat map showing ATP synthase activity across male tissues as log_2_(old/young). (D) Comparison of ATP synthase activity in young and old female tissues. Data is presented as the mean with standard error, arranged from highest (left) to lowest (right) values, and normalized to MTDR. (E) Systems-level comparison of ATP synthase activity in all 32 tissues between young and old females. (F) Heat map showing ATP synthase activity across female tissues as log2 (old/young). Statistical significance is indicated as: a = *P* < 0.05, b = *P* < 0.01, c = *P* < 0.001, d = *P* < 0.0001. Abbreviations: BAT, brown adipose tissue; gWAT, gonadal white adipose tissue; iWAT, inguinal white adipose tissue.

Compared to young males, old males showed a marked decline in CV activity in the tongue, gastrocnemius, and kidney cortex (Figure 3A). In contrast, several tissues displayed elevated CV activity with age, including the heart atrium, diaphragm, cecum, ileum, and gWAT. The low CV activity in tissues such as the pancreas, jejunum, and skin remained low and unchanged with age. The biggest change in CV activity in males was seen in striated muscles. At the systems level, the distribution of CV activity across all 32 tissues was not different between young and old males (Figure 3B). However, heatmap of CV activity based on log_2_ fold change (old/young) revealed clear tissue-level differences (Figure 3C).

Comparing young and old females showed that old females had marked reductions in CV activity in iWAT, gWAT, liver, spleen, fallopian tube, distal colon, and skin, with a corresponding increase in CV activity in the diaphragm and ileum (Figure 3D). At the systems level when all 32 tissues were compared, the CV activity distribution was not different between young and old females (Figure 3E). At the individual tissue level, however, clear differences were observed between young and old females (Figure 3F). Altogether, our data indicate age-dependent changes in CV hydrolytic activity across many tissues, with the directionality, magnitude, and tissue type differing by sex.

### 3.4 Sex differences in ATP synthase activity

**Figure 4:**
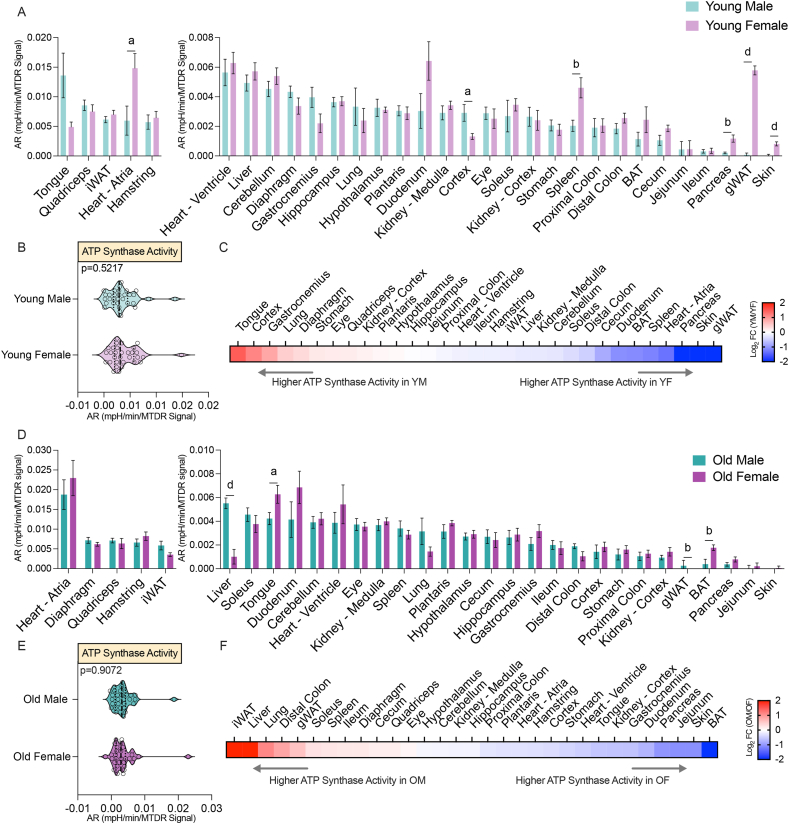
**ATP Synthase activity by sex across tissues in young and old mice**. (A) Comparison of ATP synthase activity, reflected by the acidification rate (AR), in young male and female tissues. Data is presented as the mean with standard error, arranged from highest (left) to lowest (right) values, and normalized to mitochondrial content (MTDR). (B) Systems-level comparison of ATP synthase activity in all 32 tissues between young males and young females. (C) Heat map showing ATP synthase activity across tissues as log_2_(young male/young female). (D) Comparison of ATP synthase activity in old male and female tissues. Data is presented as the mean with standard error, arranged from highest (left) to lowest (right) values, and normalized to MTDR. (E) Systems-level comparison of ATP synthase activity in all 32 tissues between old males and old females. (F) Heat map showing ATP synthase activity across tissues as log_2_(old male/old female). Statistical significance is indicated as: a = *P* < 0.05, b = *P* < 0.01, c = *P* < 0.001, d = *P* < 0.0001. Abbreviations: BAT, brown adipose tissue; gWAT, gonadal white adipose tissue; iWAT, inguinal white adipose tissue. Reproductive organs are omitted from cross-sex analysis.

In young samples, both sexes shared high CV activity in heart atrium and ventricle, striated muscles (tongue, quadriceps, hamstrings, and diaphragm), liver, cerebellum, and iWAT (Figure 4A). At the systems level, CV activity distribution was not different between the sexes (Figure 4B). However, clear sex differences were seen at the tissue level (Figure 4C). For example, young females displayed higher CV activity in the skin, gWAT, pancreas, heart atrium, and spleen, whereas young males showed higher CV activity in the tongue and cortex.

When comparing aged males and females, we found that aged males displayed lower CV activity in the tongue and BAT, and higher CV activity in the liver and gWAT (Figure 4D). At the systems level, CV activity distribution was not different between the sexes (Figure 4E). However, sex differences were seen at the tissue level (Figure 4F). Together, these data indicate that sex influences CV activity in a tissue- and age-dependent manner.

### 3.5 Age has a greater impact than sex on ATP synthase activity

**Figure 5:**
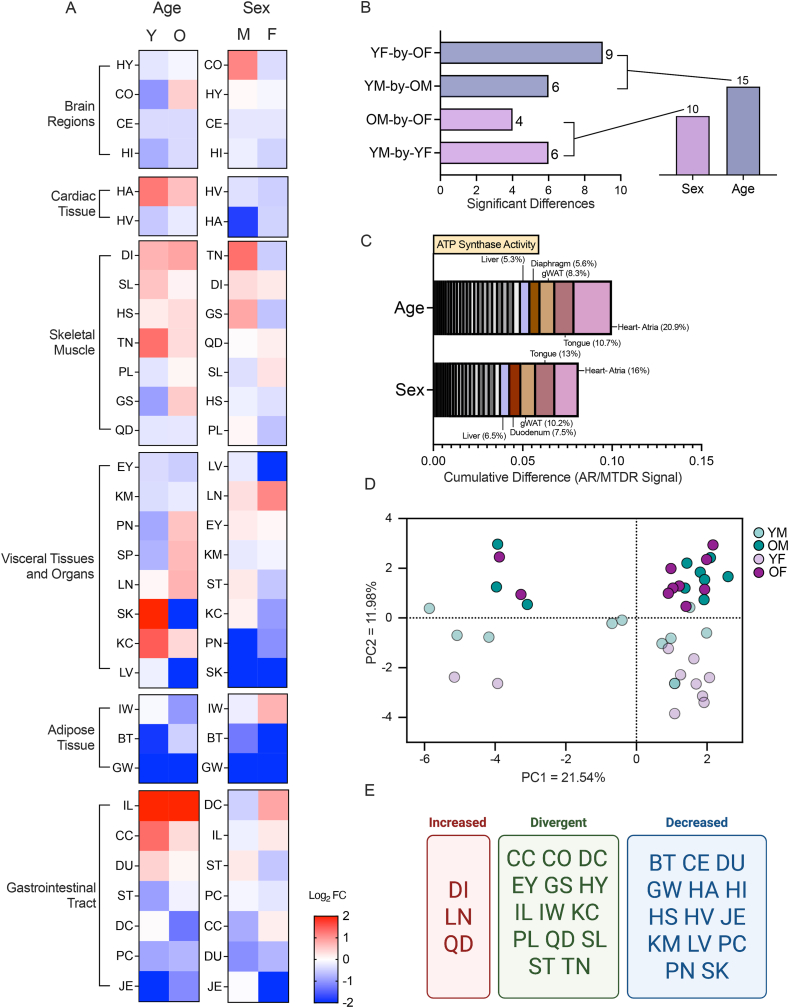
Age- and sex-associated differences in ATP synthase activity (A) Heat map showing cumulative changes between young (Y) and old (O) samples, and male (M) and female (F) samples, grouped by organ systems or tissue groups. Red color indicates changes in the positive direction (i.e., increases in ATP synthase activity) and blue color indicates changes in the negative direction (i.e., decreases in ATP synthase activity). White or closer to white color indicates no changes in ATP synthase activity. (B) Total number of significant differences across tissue-by-tissue comparisons grouped by the specific comparison and summed to the right of each histogram to highlight the number of significant findings per comparison. To underscore the number of statistically significant sex- or age-associated differences, the total number of significant findings from YM-by-YF and OM-by-OF (sex effect), and OM-by-YM and OF-by-YF (age effect) were summed. Abbreviation: YM, young male; YF, young female; OM, old male; OF, old female. (C) Cumulative absolute difference of means grouped by effect-type, sex (originating from YM-by-YF or OM-by-OF) or age (originating from OM-by-YM or OF-by-YF). These graphs do not indicate directionality of the change, only the absolute cumulative magnitude. Each box within the histogram represents a unique tissue. All histograms are organized from lowest (left) to highest (right) in degree of tissue contribution to total change given the comparison type. The top contributors to sex- or age-associated changes are highlighted with non-greyscale colors and their relative percentage of contribution to the total cumulative difference is provided. (D) Principal Component Analysis (PCA) of all tissues and groups for ATP synthase activity. (E) Summary diagrams of shared and divergent changes in ATP synthase activity across age in male and female tissues. Tissues in the red box with the labeled “Increased” have a positive change for both males and females (i.e., shared increase in ATP synthase activity). Tissues in the blue box with the label “Decreased” have a negative change for both males and females (i.e., shared decrease in ATP synthase activity). Tissues in the purple box with the label “Divergent” have opposing log_2_(O/Y) directions for males and females (i.e., changes in ATP synthase activity is opposite between males and females). Tissue abbreviation: BT = brown adipose tissue, CC = cecum, CE = cerebellum, CO = brain cortex, DC = distal colon, DI = diaphragm, DU = duodenum, EY = eyes, GS = gastrocnemius, GW = gonadal white adipose tissue, HA = heart atria, HI = hippocampus, HS = hamstring, HV = heart ventricles, HY = hypothalamus, IL = ileum, IW = inguinal white adipose tissue, JE = jejunum, KC = kidney cortex, KM = kidney medulla, LN = lung, LV = liver, PC = proximal colon, PL = plantaris, PN = pancreas, QD = quadriceps, SK = skin, SL = soleus, SP = spleen, ST = stomach, TN = tongue. Reproductive organs are omitted from cross-sex analysis.

Next, we determined the relative contribution of age and sex to CV activity. Heatmap of log_2_ fold change (old vs. young; male vs. female) grouped by organ system revealed age- and sex-specific differences in CV activity across tissues (Figure 5A). Most brain regions showed an increase in CV activity with age. In the heart, atrium showed reduced, and ventricle showed increase, CV activity with age. Skeletal muscle groups, visceral tissues and organs, and different regions of the gastrointestinal tract displayed significant variations in the directionality of change in CV activity across age and sex. Various fat depots (iWAT, gWAT) generally have lower CV activity, regardless of age and sex, except for iWAT in young females. Together, these results highlight both shared and divergent changes in CV activity across organ systems in male and female mice.

Pairwise comparisons showed that age has a larger impact on CV activity compared to sex, with a greater number of significant differences arising from old vs. young comparisons (OM vs. YM; OF vs. YF) (Figure 5B). Comparisons between sexes (YM vs. YF; OM vs. OF) yielded fewer significant differences. Summation across comparisons confirmed that age accounts for the largest number of significant differences. Cumulative absolute difference analysis also showed that age contributed the greatest impact on CV activity, with heart atrium, tongue, gWAT, and liver each accounting for 5–20% of the total cumulative shift (Figure 5C). Principal component analysis also showed a much clearer separation of samples by age than by sex (Figure 5D). Together, these results indicate a proportionally larger effect of age compared to sex on CV activity.

To provide a high-level summary view, we organized tissues into three categories based on the directionality of change in CV activity between the sexes in response to aging (Figure 5E): shared decreases, shared increases, and divergent responses. There are 14 tissues shared by males and females that show a decline in CV activity with age. We also have 14 tissues that show divergent aging responses between the sexes. In contrast, there are only 3 tissues (diaphragm, lung, and quadriceps) shared by both sexes that show an increase in CV activity with age.

### 3.6 Expression of ATP synthase related genes in mouse and human across age

**Figure 6:**
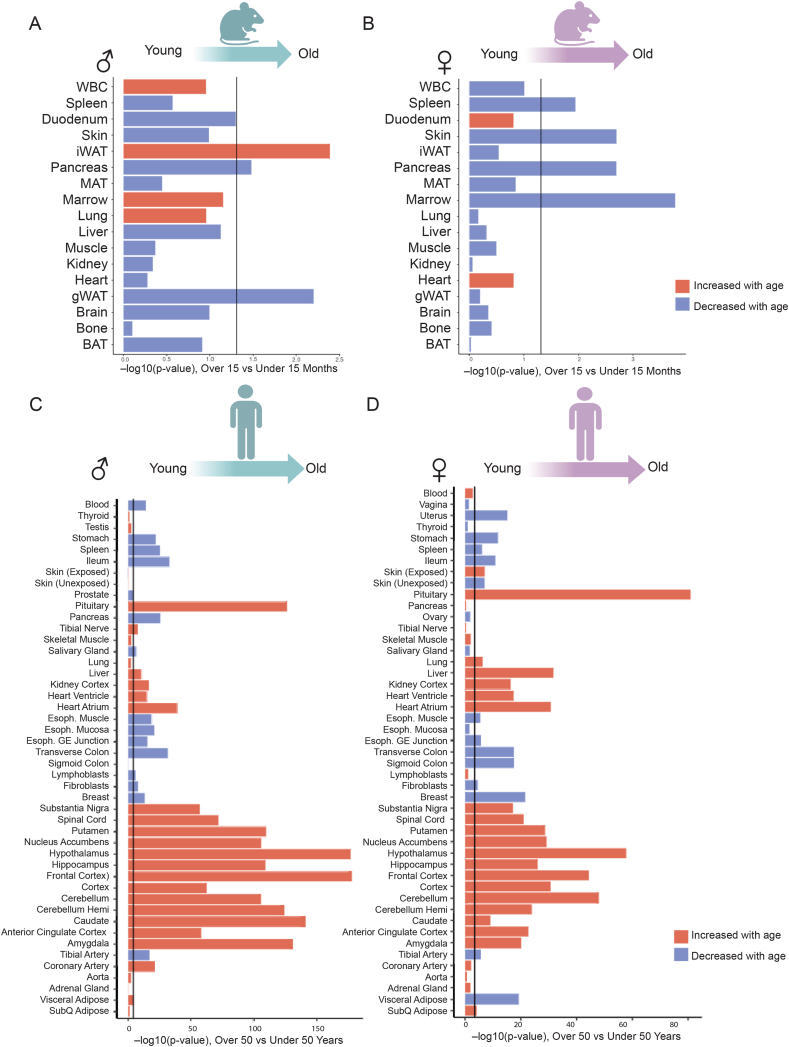
**Expression of ATP synthase related genes in mouse and human tissues across age**. Differential expression statistic [-log(pvalue)] of ATP synthase related genes (x-axis) are plotted for organs assayed (y-axis). Vertical line indicates an FDR cutoff based on number of organs being used and bar color indicates whether ATP Synthase genes increase (red) or decrease (blue) in older individuals. These analyses are shown for male mice (A), female mice (B), male humans (C) or female humans (D). Tissues included in analysis: White Blood Cells (WBC); Spleen; Bone Marrow (Marrow; femurs & tibiae); Bone (femurs & tibiae); Brown Adipose Tissue (BAT; interscapular pad); Gonadal White Adipose Tissue (gWAT); Subcutaneous White Adipose Tissue (SubQ/iWAT; inguinal pad); Mesenteric Adipose Tissue (mAT); Small Intestine – Duodenum; Pancreas; Skin (dorsal); Skeletal Muscle (Muscle; tibialis anterior); Heart; Kidney; Lung; Liver; Brain. Human tissue abbreviations: Subcutaneous Adipose (SubQ Adipose); Visceral Adipose (Adipose–Visceral, Omentum); Aorta (Artery–Aorta); Coronary Artery (Artery–Coronary); Tibial Artery (Artery–Tibial); Amygdala; Anterior Cingulate Cortex (ACC, BA24); Caudate (basal ganglia); Cerebellum; Cerebellar Hemisphere; Cortex; Frontal Cortex (Frontal Ctx, BA9); Hippocampus; Hypothalamus; Nucleus Accumbens (basal ganglia); Putamen (basal ganglia); Spinal Cord (C1, cervical); Substantia Nigra; Tibial Nerve; Sigmoid Colon; Transverse Colon; Esophagus – Gastroesophageal Junction (Esoph. GE Junction); Esophagus – Mucosa (Esoph. Mucosa); Esophagus – Muscularis (Esoph. Muscle); Small Intestine – Terminal Ileum (Ileum); Liver; Pancreas; Salivary Gland (Minor); Stomach; Adrenal Gland; Pituitary; Thyroid; Heart Atrium (Atrial Appendage); Heart Ventricle (Left Ventricle); Whole Blood (Blood); Spleen; Cultured Fibroblasts (Fibroblasts); EBV-transformed Lymphoblasts (Lymphoblasts); Skeletal Muscle; Lung; Skin (Unexposed, suprapubic); Skin (Exposed, lower leg); Mammary Tissue (Breast). Male reproductive tissues: Prostate; Testis. Female reproductive tissues: Ovary, Uterus, and Vagina.

Lastly, we analyzed human [21] and mouse [15] tissue-wide transcriptome data to determine whether changes in CV activity across age and sex are due to differences in the expression of CV related genes. While expression of CV related genes (19 in total) in several tissues changed regardless of sex (e.g., different brain regions in humans), most organs displayed a sex-dependent expression pattern based on age (Figure 6). In mouse tissues, aging was predominantly associated with transcriptional downregulation of CV related genes in both sexes (Figure 6A–B). In humans, aging had variable effects on CV related gene expression, where many peripheral tissues showed reduced expression while most brain regions showed increased expression (Figure 6C–D). In both human and mice, we observed only partial concordance between CV gene expression and CV hydrolytic activity. For example, in mice we saw concordance in gWAT, BAT, liver, and brain for males and concordance in spleen, pancreas, liver, skin, lung, iWAT, gWAT, and BAT for female. In humans, we saw fewer concordance across tissues. Together, these results suggest that changes in CV activity with age are likely modulated by a combination of transcriptional and posttranscriptional mechanisms.

## 4 Discussion

In this study, we applied an approach recently developed by Fernandez-Del-Rio and co-workers [20] to comprehensively profile ATP synthase hydrolytic activity across tissues, sex, and age. This approach allows the maximal hydrolytic capacity of ATP synthase to be directly quantified in tissue homogenates without the need for mitochondrial isolation and minimal disruption (i.e., no detergents used), and also does not rely on indirect chemical coupling reactions [[24], [25], [26], [27], [28]]. This method is robust, reproducible, and can be carried out at scale on a large number of previously frozen tissue samples. By applying this standardized method to 1280 tissue samples on a single common platform (Seahorse XFe96 Analyzer), we were able to generate a high-quality ATP synthase activity atlas spanning 32 tissues from young and old male and female mice, thus providing a global view of ATP synthase function across tissues, sex, and age.

Our atlas shows that the hydrolytic activity of ATP synthase across tissues is significantly modulated by sex and age—the two major biological variables known to affect organismal health and the functional trajectory of organ systems over time. While sex differences in CV activity were clearly observed, age has a much larger impact on CV activity across tissues. We observed proportionately more tissues whose CV activity is affected by age than by sex, a phenomenon also seen when we profiled mitochondrial respiration [19]. Indeed, the dominance of age over sex in dictating mitochondrial function is much more striking for mitochondrial respiration than for CV activity.

Male and female mice respond very differently to aging, both in tissue type and in the magnitude and directionality of change in CV activity. In males, CV activity is markedly reduced in 3 tissues (tongue, gastrocnemius, and kidney cortex) but strikingly elevated in 5 tissues (heart atrium, diaphragm, cecum, ileum, and gWAT). In females, by contrast, CV activity is markedly reduced in 7 tissues (iWAT, gWAT, liver, spleen, distal colon, skin, and fallopian tube) but increased in 2 tissues (diaphragm and ileum). Of all the age-associated changes in CV activity, only two tissues are shared by males and females (i.e., diaphragm and ileum). There are more than twice as many tissues in females than males where we observed a decline in CV activity with age, suggesting greater vulnerability in females to age-induced functional changes in ATP synthase. In contrast, there are more than twice as many tissues in males where we observed a significant increase in CV activity, suggesting that males respond very differently to age-associated changes in CV function. Although evidence is lacking, we speculate that elevated CV activity may reflect a compensatory response to declining mitochondrial function with age.

Other sex differences in CV activity are also evident, likely reflecting the impact of sex steroids. Estrogen is known to promote mitochondrial biogenesis through a transcriptional network involving PGC-1α, NRF1/2, and TFAM [12,29], while testosterone is known to act in skeletal muscle and other peripheral tissues to enhance anabolic signaling and mitochondrial function [30]. Compared to young males, young females have markedly higher CV activity in the heart atrium, spleen, pancreas, gWAT, and skin. Further, in tissues such as the tongue, gastrocnemius, and kidney cortex, females are able to maintain similar levels of CV activity with age while CV activity declines significantly in the same tissues in males. We speculate that altered sex steroid levels with age may account, in part, for the differences seen in CV activity across tissues and sex. We also noted interesting patterns of CV activity in the reproductive tissues. For example, CV activity remains unchanged in testes and declined in fallopian tube with age, perhaps reflecting declining estrogen actions in the female reproductive tissues [29].

Adipose tissues also show interesting depot- and sex-specific changes in CV activity with age. In young mice, females have much higher CV activity in gWAT than males. In aged animals, females have higher CV activity in BAT and lower activity in gWAT than males. In response to aging, females have markedly reduced CV activity in iWAT and gWAT, whereas males have greatly elevated CV activity in gWAT. The divergent changes in CV activity may reflect age- and sex-associated differences in sympathetic innervation, adiposity and lipid turnover, and inflammatory signaling within fat depots [31,32].

The differences in CV activity across tissues, age, and sex can only be partially attributed to differences in the expression of ATP synthase related genes. This is not unexpected as most biological processes are regulated by a combination of transcriptional and posttranscriptional mechanisms. However, there are several caveats associated with our transcriptomic analysis. While the human transcriptome data [21] from GTEx contain a relatively large sample size, the mouse transcriptome data [15] contain only 4 males and 2 females per time point (with a total of 10 time points across the lifespan). In our study, we chose 50 years of age in humans and 60 weeks in mice as cutoffs for defining young versus old. Given that aging is a continuous process and organ function declines progressively over time, additional studies involving much larger sample size across age are warranted.

We wish to highlight several limitations of our study. Given the extensive cost and labor associated with the present study, only two major time points (10 and 80 weeks old) were chosen for the quantification of tissue ATP synthase activity. The inclusion of additional time points (e.g., 40 and 120 weeks old) in future studies will likely provide greater temporal resolution of ATP synthase activity across the lifespan. Our assay quantifies maximal ATP synthase hydrolytic capacity (the reverse mode) rather than ATP synthesis (the forward mode). Although ATP hydrolysis provides a surrogate measure of CV catalytic function [20], it does not capture the balance of ATP synthesis versus hydrolysis that occurs under physiologic conditions. Our method focuses only on the catalytic activity of ATP synthase; however, in an intact mitochondrion, ATP synthase activity is closely linked to the electrochemical proton gradient across the inner mitochondrial membrane. The use of frozen tissue homogenates makes it possible to determine ATP synthase activity at scale in a large number of tissues, but the procedure—despite minimal disruptions and no detergents used—necessarily disrupts mitochondrial integrity. Given that cristae architecture remodels with age, and that cristae remodeling influences ATP synthase dimerization, proton flux efficiency, and the switch between ATP synthesis and hydrolysis [33], our measurements may underestimate the changes in CV activity with age. Lastly, all organs and tissues contain multiple cell types and the method we used fails to capture changes in CV activity at cellular resolution. Single-cell transcriptomic data of CV related genes may provide some insights, but our analysis of bulk RNA-seq data from humans and mice suggests that gene expression changes may only partially associate with changes in CV activity. We anticipate future technological innovations may allow for large-scale determination of CV activity at single-cell resolution across tissues.

In summary, our atlas provides a systems view of ATP synthase activity across tissues, sex, and age. The atlas provides a valuable reference for the mitochondrial community, helping to distinguish aging-versus disease-associated functional changes in ATP synthase function. The atlas is also valuable for informing studies on mitochondrial adaptation across various physiological and disease states.

## CRediT authorship contribution statement

**Muzna Saqib:** Conceptualization, Data curation, Formal analysis, Investigation, Methodology, Visualization, Writing – original draft. **Dylan C. Sarver:** Conceptualization, Investigation, Methodology, Writing – review & editing. **Christy M. Nguyen:** Formal analysis, Methodology, Visualization, Writing – review & editing. **Fangluo Chen:** Investigation, Writing – review & editing. **Marcus M. Seldin:** Data curation, Formal analysis, Methodology, Supervision, Visualization, Writing – review & editing. **G. William Wong:** Conceptualization, Formal analysis, Funding acquisition, Investigation, Project administration, Supervision, Writing – original draft.

## Declaration of competing interest

None.

## Data Availability

Data will be made available on request.

## Appendix A Supplementary data

Supplemental Table 1 Supplemental table with all ATP synthase activity data for all tissues in young and old male and female mice. Supplemental Table 1
